# Retinal manifestations of traumatic brain injury

**DOI:** 10.1038/s41598-025-94091-1

**Published:** 2025-04-29

**Authors:** Elinor Laws, Youstina Metry, Noor Haziq Bin Saliman, Antonio Belli, Richard J. Blanch

**Affiliations:** 1 Ophthalmology Department, University Hospitals Birmingham NHS Foundation Trust, Birmingham, UK; 2 Neuroscience and Ophthalmology, School of Infection, Inflammation and Immunology, College of Medical and Dental Sciences, University of Birmingham, Birmingham, UK; 3 NIHR Surgical Reconstruction and Microbiology Centre, University Hospitals Birmingham NHS Foundation Trust, Birmingham, UK; 4 NIHR Birmingham Biomedical Research Centre, University of Birmingham, Birmingham, UK; 5 Centre for Optometry Studies, Faculty of Health Sciences, Universiti Teknologi MARA Cawangan Selangor, Bandar Puncak Alam Selangor, Malaysia; 6 Neurosurgery Department, University Hospitals Birmingham NHS Foundation Trust, Birmingham, UK; 7 Academic Department of Military Surgery and Trauma, Royal Centre for Defence Medicine, Birmingham, UK

**Keywords:** Brain injuries, Cell death in the nervous system, Diagnostic markers

## Abstract

Retinal nerve fibre layer (RNFL) and ganglion cell layer (GCL) thinning occur weeks to months after traumatic brain injury (TBI), even without computed tomography (CT) findings. The patterns of RNFL and GCL loss and their relationship to TBI severity and CT findings have not been characterised. This observational study included consecutive patients assessed in hospital after TBI. All patients underwent OCT. A literature review was conducted to determine the test–retest variability of RNFL and GCL measurements. Of 135 included patients, 62 had follow up OCTs. The test–retest limit of agreement for global RNFL thickness was 4 µm. Two patients had symptomatic traumatic optic neuropathy, 17 had less severe RNFL thinning on follow up, six RNFL thickening and 31 no RNFL changes. Higher TBI severity, Marshall CT classification and lower time to first OCT after injury strongly associated with subsequent RNFL changes (p < 0.001 for all). Global RNFL thickness in patients with initial OCT < 42 days after injury declined by 1.74 µm/month with Marshall II CT findings, compared 0.05 µm/month with Marshall I, and 3.69 µm/month after severe TBI, versus 1.47 µm/month after mild. Subclinical OCT changes therefore occur after TBI, and may contribute to future multimodal TBI diagnostic and severity assessments.

## Introduction

Traumatic Brain Injury (TBI) is common, affecting an estimated 69 million individuals each year globally^1^, and causing an estimated 1.4 million emergency department visits annually in the UK, of which 200,000 require hospital admission^2^. Sports injury, falls, assault and road traffic collisions are the most common causes of TBI in the civilian population, and the incidence is increasing with 12% more admissions in 2019 than 2014^3^. The high rates of morbidity and mortality after TBI place a significant burden on the global health economy^1^.

TBI typically presents with loss of consciousness, amnesia, and neurological dysfunction^4,5^. Severity is affected by the method of injury and whether impact was direct or indirect ^6^, and is classified by symptoms and signs, for example, using the Department of Defence (DoD) and Mayo criteria, as well as by computed tomography (CT) head findings using the Marshall score (Supplementary Table S1)^7–9^. One limitation of the classification systems is that many TBI patients with significant symptom burden show no abnormality on CT, creating clinical complexity in diagnosis and management^10^.

Traumatic optic neuropathy (TON) is a clinical syndrome caused by TBI where CT may be normal even when vision is severely affected. Occurring in around 0.5–8% of TBI cases, TON is characterised by sudden visual loss after trauma, with a relative afferent pupillary defect in unilateral cases^11^. Patients with TON may undergo investigation using optical coherence tomography (OCT) retinal imaging, which is usually normal soon after injury, as retinal ganglion cell layer (GCL) and retinal nerve fibre layer (RNFL) thinning occur weeks to months later^12,13^.

The brain and retina share embryological origins and autoregulatory functions^14,15^, and neurological conditions such as stroke, Parkinson’s disease, multiple sclerosis, and Alzheimer’s dementia are associated with changes in retinal structure^16,17^, which may have diagnostic value^18^. Altered retinal structure is therefore expected after acute TBI, both in the context of TON and reflecting wider loss of brain neurons. TBI has two phases: primary injury occurs at the time of impact, whilst secondary injury occurs as a result of ischaemia and inflammation, causing delayed cell death. Both primary and secondary injury may affect the optic nerve, but only primary visual loss at the time of trauma has been reported with the exception of progressive loss of retinal nerve fibres and visual function reported years after injury by Gilmore et al.^19,20^.

RNFL changes occur in 30% of patients with mild TBI in the single study that has reported this statistic, with visual field deficits in 40%^21^, while another study reported disc pallor in 47% ^22^, similar to our previously reported rate of GCL thinning of 43% after moderate to severe TBI^12^. The patterns of retinal ganglion cell (RGC) alterations, and their relationship to TBI severity or CT findings have not been categorised. We therefore conducted a study to categorise OCT changes after TBI, across the range of injury severity, and explore their relationship with brain imaging findings.

## Methods

Consecutive patients were prospectively included in a service evaluation of eye assessments after TBI at our tertiary trauma hospital from March 2020 to May 2022. Patients were over 16 years of age, and only included if they were able to cooperate with testing as part of routine clinical care, and timing of these tests varied between patients according to their individual clinical need. Retrospective use of clinical service data for this study was approved by the Health Research Authority (23/HRA/0136) and the Research Governance Department of University Hospitals Birmingham NHS Foundation Trust, including a determination that consent was not required, as no patient-identifiable information were processed outside of the clinical care team, and was performed in accordance with the Data Protection Act (2018).

Data collected for all patients were: age at time of injury, sex (assigned at birth), visual acuity, OCT images, date of injury, method of injury, CT findings with Marshall Score and TBI severity by the DoD classification^7,9^. Time to first OCT was assessed as the number of days between the injury and the first OCT examination. TBI data were collected for the most recent TBI where patients had experienced multiple events. The DoD classification takes into account duration of loss of consciousness and alteration in mental state, duration of post-traumatic amnesia, Glasgow coma score assessment at time of injury and structural abnormalities on brain imaging (either CT or MRI) to determine whether the TBI is mild, moderate, or severe (Supplementary Table S1).

**Fig. 1:**
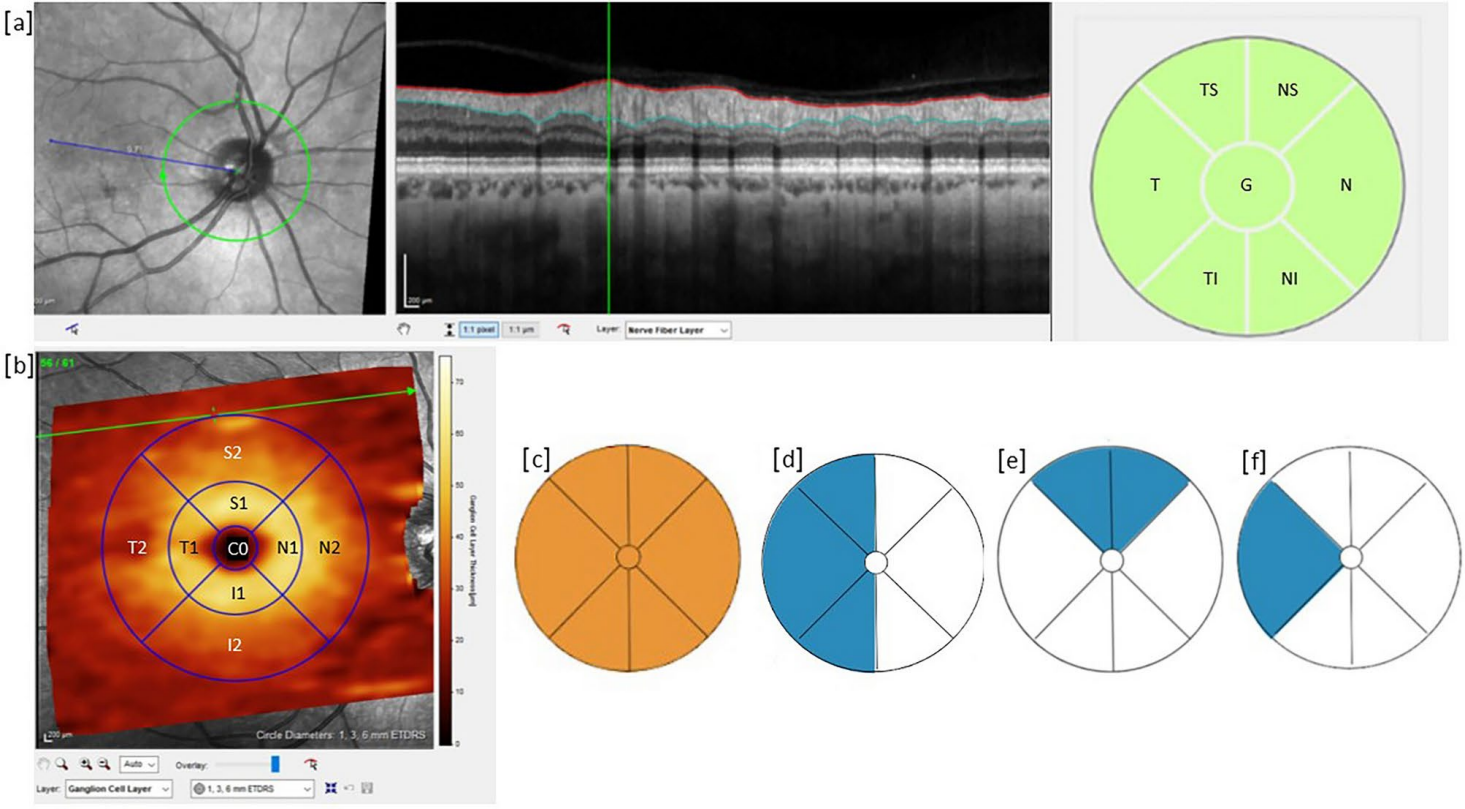
(**a**-**b**). Illustrative retinal nerve fibre layer (RNFL) (**a**) images showing segmented global (G) and sectoral thickness in six sectors: N (nasal), T (temporal), NS (superonasal), TS (superotemporal), NI (inferonasal), and TI (inferotemporal). Illustrative ganglion cell layer (GCL) image (**b**), showing segmented sectoral thickness in ETDRS (Early Treatment of Diabetic Retinopathy Study) grid areas; C0 (foveal), S1 (inner, superior), S2 (outer, superior), T1 (inner, temporal), T2 (outer, temporal), I1 (inner, inferior), I2 (outer, inferior), N1 (inner, nasal), N2 (outer, nasal. (**c**-**f**) Illustrative archetype RNFL changes: (**c**) traumatic optic neuropathy, RNFL thinning in all sectors and global loss > 20 µm; (**d**,**e**) multisectoral RNFL thinning; (**f**) single sectoral RNFL thinning.

All OCTs were acquired using SPECTRALIS® Heidelberg OCT2 table-top module (Heidelberg Engineering, Heidelberg, Germany). Measurements of GCL thickness in the macula, and RNFL thickness around the optic nerve head, were obtained using manufacturer’s software (Heidelberg Eye Explorer, Version 1). Peripapillary RNFL thickness was measured using the RNFL protocol, which generates a map of the average thickness (Global—G0), and six sectors thickness N (nasal), T (temporal), NS (superonasal), TS (superotemporal), NI (inferonasal), and TI (inferotemporal), as shown Fig. 1a. Macula OCT volumes were obtained using the manufacturer’s ‘Posterior Pole’ protocol acquiring 61 horizontal B-scans averaged over at least 9 frames in a 30° × 25° fovea-centred volume. The macula was divided into ETDRS (Early Treatment of Diabetic Retinopathy Study) grid areas; C0 (foveal), S1 (inner, superior), S2 (outer, superior), T1 (inner, temporal), T2 (outer, temporal), I1 (inner, inferior), I2 (outer, inferior), N1 (inner, nasal), N2 (outer, nasal; Fig. 1b). All OCT images and segmentation were verified by EL and YM, with disagreements resolved by RB. OCT thickness measurements were compared between initial assessment and follow-up.

To determine the limits of test–retest variability in RNFL and GCL layers, when using Heidelberg Spectralis OCT, published literature was identified by PubMed, Medline and Google Scholar searches on September 1^st^, 2023, using the search terms: [[reproducibility] OR [repeatability]] AND [Heidelberg] AND [[RNFL] OR [retinal nerve fibre layer] OR [GCL] OR [ganglion cell layer]] and reference lists were additionally reviewed. Titles and abstracts were reviewed by YM, full papers reviewed, and data extraction performed by YM and RJB. TON was defined as global change > 20 µm in RNFL (when follow up OCT was available) with reduced visual acuity and a RAPD where unilateral^23–25^.

OCT changes were classified into archetypes according to pattern of thinning, thickening or both (Fig. 1c-f).

### Statistical analysis

Data were analysed in SPSS v29 (IBM, Armonk, NY, USA) using Generalised Estimating Equations, with a linear model, and treating continuous measures (age and time) as covariates and categorical variables as factors, with eye and retinal area treated as within-subjects repeated measures. For illustrative purposes time to first OCT was grouped into three groups: < 42 days, 42–150 days, > 150 days. To address the variable time between initial and follow up OCT, RNFL changes were modelled as change per month. Categorical analysis used Chi-squared. Unless otherwise specified, data are presented as mean ± standard error of the mean.

## Results

Patient demographics are shown in Table 1. A total of 135 patients were included in the study, and 62 (45.93%) patients had more than one (follow up) OCT, of whom 35.5% had mild, 46.8% moderate, and 17.7% severe TBI, compared to 81% mild, 9% moderate and 10% severe TBI amongst patients who did not attend for follow up OCT (p < 0.001).

**Table 1:** Patients’ demographic and baseline data. *RE* right eye, *LE* left eye, *RTC*, road traffic collision.

Age	Mean: 31 years Median: 27 years Range 16–82 years
Sex	Male: 109 (81%) Female: 26 (19%)
Initial LogMAR VA LogMAR (Snellen)	Median: RE -0.1 (20/16) LE -0.04 (20/20)
Follow up LogMAR VA LogMAR (Snellen)	Median: RE -0.04 (20/20) LE -0.06 (20/16)
Mechanism of injury	Sport: 76 (56%) RTC: 20 (15%) Assault: 16 (12%) Fall: 23 (17%)
Multiple TBI in lifetime	29/135 (21%)
TBI severity	Mild: 81 (60%) Moderate: 36 (26.7%) Severe: 18 (13.3%)
Mean time to initial OCT after TBI for patients with follow up OCT assessments (S.D.)	Mean: 50.9 days (80.7)
Follow up OCT	Total: 62/135 (45.9%)
Mean time to follow up OCT after TBI (S.D.)	Mean: 157.3 days (152.4)
CT head	Total: 60/135 (44.44%)
Marshall classification score	I: 10 (7%) II: 35 (26%) III: 6 (4%) IV: 0 V: 9 (7%) VI: 0
MRI head	54/135 patients (40%) With findings: 19 (35%)

Images from three patients were excluded, one as their initial OCT showed papilloedema that resolved on follow-up (causing significant RNFL thinning compared to the initial OCT), the second patient had high myopia associated with peripapillary retinoschisis making segmentation unreliable, and the third, as time to initial TBI was an outlier (13 years). GCL data for 3/62 patients were excluded due to imaging artefacts causing unreliable segmentation.

Forty-four percent of patients had CT head (88.3% of moderate-severe TBI, 11.7% mild TBI). For analysis of CT findings, patients were grouped as: 10 patients (16.67%) Marshall score I, 35 patients (58.3%) Marshall score II and 15 patients (25%) Marshall score of III or above (Table 1).

Forty-six (34%) participants were recorded as reporting visual disturbance on initial presentation or follow-up (photophobia 15.6%, blurred vision 15.6%, field defect 4.4%, diplopia with cranial nerve III, IV, or VI palsy 2.2%, diplopia without cranial nerve palsy 1.5%).

### Limits of agreement for RNFL and GCL changes

Published test–retest variability for RNFL thickness measurements using Heidelberg OCT are summarised in Table 2. Published test–retest variability for GCL thickness measurements using Heidelberg OCT are summarised in Table 3.

**Table 2:** 95% limit of agreement for retinal nerve fibre layer (RNFL) test–retest variability (thicknesses in µm).* HC* healthy control patients,* Gl* glaucoma patients,* DM* diabetes mellitus without diabetic retinopathy,* PP* pseudophakic,* n* number of patients,* N* nasal,* T* temporal,* NS* superonasal,* TS* superotemporal,* NI* inferonasal,* TI* inferotemporal,* PMB* papillomacular bundle,* NK* not known.

RNFL sector	Garcia Martin 2011 ^26^	Tan 2012 ^27^	Wu 2011 ^28^		Langenegger 2011 ^29^		Garcia-Martin 2013 ^30^		Bambo 2014 ^31^		Kochendorfer 2014 ^32^		Limits of agreement based on published data
	HC n = 61	HC n = 50	HC n = 45	Gl n = 33	Gl n = 47	HC n = 56	Cataract + DM n = 35	PP + DM n = 35	Cataract n = 60	PP n = 60	HC n = 36	Gl n = 26	
Global (µm)	3.62	3.5	2.63	2.23	1.86	1.9	4.74	3.79	5.53	3.72	1.86	1.47	≥ 4
NS (µm)	12.9		3.67	4.41	5.35	4.54	10.2	9.13	10.1	9.98	3.53	2.25	≥ 10
S (µm)		6.41	3.78	3.41			7.94	6.89	13.1	8.17	3.16	1.81	
TS (µm)	10.4		4.68	3.43	3.48	3.65	9.17	8.83	7.82	8.69	3.59	1.89	≥ 9
T (µm)	5.62	6.16	3.59	3.39	1.96	2.13	6.12	5.71	4.64	5.72	2	2.13	≥ 6
TI (µm)	8.02		4.68	4.08	6.41	3.87	9.88	9.61	7.78	11.0	4.11	2.16	≥ 10
I (µm)		5.4	3.8	3.27			6.9	4.92	5.64	9.46	3.32	1.65	
NI (µm)	10.2		3.59	3.45	3.47	8.54	11.4	9.78	10.4	11.9	4.61	2.28	≥ 10
N (µm)	9.13	3.55	3.33	2.9	3.38	3.56	6.7	5.81	8.53	7.24	3.05	2.77	≥ 8
PMB (µm)					2.87	3.3							
InI (days)	< 1	NK	< 1	< 1	1	1	NK	NK	< 1	< 1	< 1	< 1

**Table 3:** 95% limit of agreement for ganglion cell layer (GCL) thickness test–retest variability (thicknesses in µm). HC, healthy control patients; InI, interscan interval; n, number of patients; I1, inner inferior; I2, outer inferior; N1, inner nasal; N2, outer nasal; S1, inner superior; S2, outer superior; T1, inner temporal; T2, outer temporal.

GCL sector	Çetinkaya 2017 ^33^	Ctori 2015 ^34^	Jimenez Santos 2021 ^35^	Summary limits of agreement based on published data
	HC n = 60	HC n = 40	HC n = 79	
Fovea (central; µm)	7.72	3.11	1.99	≥ 5
Inner Circle (µm)	7.72	5.04		
N1 (µm)			3.87	≥ 5
T1 (µm)			3.77	≥ 5
S1(µm)			3.45	≥ 5
I1 (µm)			3.81	≥ 5
Outer circle (µm)	6.60	10.6		
N2 (µm)			1.98	≥ 7
T2 (µm)			2.48	≥ 7
S2 (µm)			2.5	≥ 7
I2 (µm)			3.29	≥ 7
InI (days)	< 1	< 1	< 1	

### Cross-sectional analysis of OCT findings after injury

Across the whole patient cohort of 135 patients, looking at the RNFL thickness on the first (or only) OCT, there was no evidence that the 21% who reported multiple TBI had different RNFL thicknesses from those who did not (p = 0.321), or that time after injury of OCT assessment affected RNFL thickness (p = 0.908).

### OCT changes at follow up, and their association with TBI severity and CT findings

**Fig. 2:**
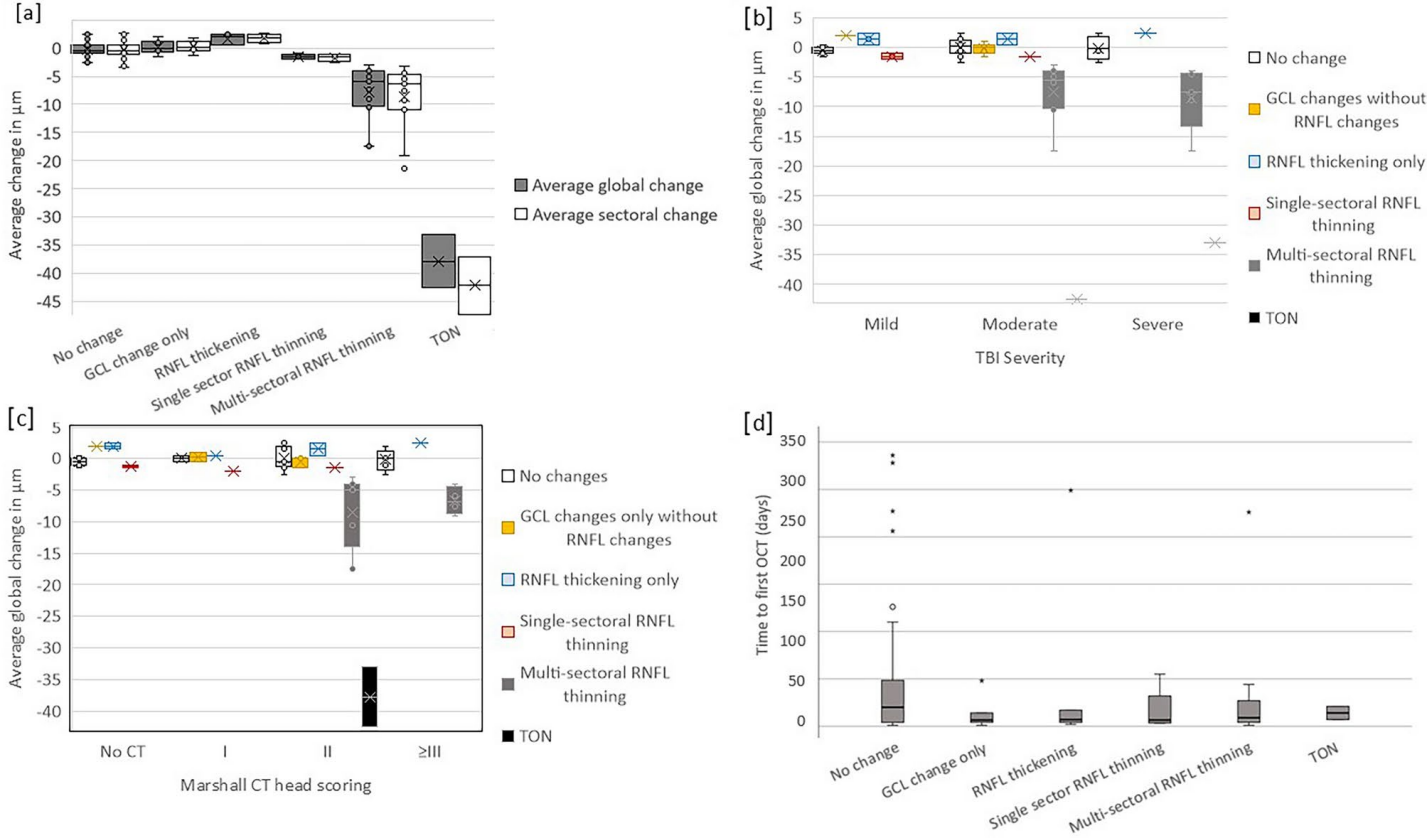
Boxplots showing: (**a**), average global retinal nerve fibre layer (RNFL) thickness changes on follow-up; (**b**), association between average global RNFL thickness changes on follow up and traumatic brain injury (TBI) severity; (**c**), association between average global RNFL thickness changes on follow up and Marshall score; (**d**), association between average time from injury to first optical coherence tomography (OCT) imaging and RNFL thickness change on follow up.* GCL* ganglion cell layer.

Thirty-one (31/62; 50%) patients had RNFL or GCL changes based on the defined limits of agreement (Tables 2–3), of whom 11/31 had visual symptoms. RNFL changes were grouped into single sector RNFL thinning, multi-sector RNFL thinning, RNFL thickening, TON, isolated GCL thinning or thickening, and no RNFL or GCL changes (Fig. 1c-f). RNFL thickness changes were more marked than GCL changes (Table 4, Fig. 2A and Supplementary Table S2), with TON and multisectoral RNFL thinning more associated with moderate to severe TBI and RNFL thickening with mild to moderate TBI.

**Table 4:** Initial and follow-up ganglion cell layer (GCL) and retinal nerve fibre layer (RNFL) thickness in µm. p values given for the 2-tailed comparison between initial and follow-up OCT (t-test). GCL: C0, Fovea, *I1* inner inferior, *I2* outer inferior, *N1* inner nasal, *N2* outer nasal, *S1* inner superior, *S2* outer superior, *T1* inner temporal, *T2* outer temporal. *RNFL N* nasal, *T* temporal, *NS* superonasal, *TS* superotemporal, *NI* inferonasal, *TI* inferotemporal.

	Sector	Initial: all patients (n = 132 GCL, n = 135 RNFL)	SE	Initial: patients with follow-up (n = 59 GCL, n = 62 RNFL))	SE	Follow-up	SE	p-value
Mean (S.D.) GCL thickness (µm)	C0	14.2 (4.18)	0.26	14.0 (3.87)	0.35	13.6 (3.92)	0.36	0.005
	N1	52.3 (6.69)	0.41	50.8 (7.73)	0.71	49.9 (8.98)	0.82	0.044
	T1	49.3 (6.99)	0.43	47.3 (7.76)	0.71	46.7 (9.07)	0.83	0.175
	S1	53.9 (6.02)	0.37	52.5 (6.65)	0.61	52.0 (7.91)	0.73	0.24
	I1	53.4 (6.53)	0.4	51.8 (7.48)	0.68	51.1 (9.16)	0.84	0.132
	N2	39.8 (4.01)	0.25	39.4 (4.18)	0.38	39.0 (5.15)	0.47	0.235
	T2	37.5 (4.65)	0.29	36.5 (5.09)	0.47	36.1 (5.76)	0.53	0.227
	S2	36.1 (3.51)	0.22	35.8 (3.75)	0.34	35.6 (4.45)	0.41	0.357
	I2	34.8 (3.63)	0.22	34.5 (3.97)	0.36	34.3 (4.46)	0.41	0.263
Mean (S.D.) RNFL thickness (µm)	Global	101 (9.85)	0.6	101 (10.1)	0.9	97.9 (12.47)	1.12	0.001
	N	77.6 (14.37)	0.88	76.7 (13.97)	1.25	74.5 (15.06)	1.35	0.001
	T	69.4 (11.19)	0.68	69.9 (11.68)	1.04	67.8 (12.99)	1.16	0.004
	NS	113 (23.96)	1.46	116 (27.23)	2.44	112 (24.08)	2.15	0.002
	TS	141 (20.02)	1.22	140 (20.08)	1.8	135 (21.73)	1.94	0.003
	NI	116 (23.11)	1.41	115 (24.78)	2.22	112 (26.64)	2.38	0.002
	TI	142 (19.79)	1.21	142 (21.35)	1.91	139 (24.83)	2.22	0.004

Five patients out of 62 (8.1%) developed homonymous GCL thinning suggestive of optic tract injury, classified as multi-sectoral RNFL thinning (4 patients) and TON (1 patient) and associated with corresponding visual field changes in three of the four patients who also had Humphrey visual field (HVF) assessment.

TBI severity and Marshall score both strongly associated with RNFL thickness changes in both global and individual sectoral measures (Fig. 2b and 2c; p < 0.001 for all). Time to first OCT was also strongly associated with change in RNFL thickness (p < 0.001), with the greatest changes seen in global RNFL thickness in patients with Marshall II findings on CT with initial OCT less than 42 days (-1.74 µm / month ± 0.35 µm) and 42–150 days (-2.57 µm / month ± 1.30 µm), compared to patients with Marshall 1 (-0.05 µm / month ± 0.40 µm) and Marshall III or more (-0.70 µm / month ± 0.35 µm) in patients seen less than 42 days after injury. Similarly, patients with severe TBI with initial OCT less than 42 days (-3.69 µm / month ± 0.65 µm) and OCT between 42 and 150 days (-2.81 µm ± 1.98 µm) had greater thinning than patients with severe TBI seen 42–150 (-2.81 µm / month ± 1.98 µm) and more than 150 days (-0.987 µm / month ± 0.56 µm) after injury or patients with mild TBI seen within 42 days (-1.47 µm / month ± 0.63 µm). Women had 1.08 ± 0.44 µm / month less RNFL thinning than men on average (p = 0.015). There was no differential RNFL change by sector (p = 0.181) and no association between age (p = 0.105), or multiple previous TBI (p = 0.098) and RNFL change.

There was no association between Marshall score or TBI severity and GCL changes (p = 0.118 and p = 0.209 respectively) and no differential changes in patients with initial OCT at different times after injury (TBI, p = 0.542; Marshall score, poor model fit).

### Visual loss in patients with TON

Two patients (1.5%) had TON with visual loss and RNFL thinning of more than 20 µm on follow up and corresponding GCL loss. On initial and follow-up assessments of visual acuity, one had LE visual acuity of counting fingers (CF), and one had LE no perception of light (NPL) with no recovery.

## Discussion

We report, for the first time, an association between OCT RNFL thinning on longitudinal follow up after TBI and TBI severity and CT brain imaging changes, with more severely injured patients suffering a greater degree of RNFL loss. TON occurred in 1.5% of all patients, but of those with OCT follow-up, 46.8% had changes that were detectable on OCT but otherwise subclinical. A high number of patients had changes within the expected test–retest variability, and when considering the group as a whole, the changes varied by time after TBI and by injury severity.

Indirect TON is classically described as involving a relative afferent pupillary defect (in unilateral cases), loss of colour vision and variable loss of visual acuity and visual field and subsequent optic atrophy after TBI^36^. The incidence is not clearly reported in literature, with reported incidence rates between 0.5 and 8% of all patients with TBI^11^. Our results are consistent with prior case series, with two cases of traumatic optic neuropathy fitting the classical definition at initial assessment (1.5% of the 135 patients assessed after TBI).

It is clear however, that many patients do not meet the classical diagnostic criteria for TON, but do suffer subclinical TON, with RNFL changes that may be multisectoral, single sector or global thinning or, in a small proportion of cases, manifest as thickening. We report OCT RNFL changes in 50% of our cohort, and isolated GCL changes (without detectable RNFL change) in an additional six patients (9.68%). Mixed thickening and thinning was observed in Olympic boxers over 18 months^37^, and we have previously reported GCL thinning in 43% of patients with moderate to severe TBI^12^, and RNFL thinning is reported in 30% of mild TBI patients^11,13,21^, all consistent with our findings. Retinal thickening occurs after hypoxia, for instance in patients infected with COVID-19^38^, and mouse models^39,40^, and retinal gliosis occurs in the mouse blast injury traumatic optic neuropathy model^41^, although the determinants of whether the retina thickens or thins are unclear.

Despite most patients lacking detectable RNFL changes at initial assessment, global and sectoral RNFL thinning at follow up were both strongly associated with both TBI severity and Marshall score, suggesting that RNFL change after TBI represents a continuous spectrum, from TON to subtle changes that are less than the normal test–retest variability. In most cases changes were less than normal inter-person variation in the population, meaning that they can only be detected by repeated measurements to detect change over time. RNFL and GCL loss after TBI may therefore be an expected finding, which does not necessarily associate with symptomatic visual loss, but may associate with TBI severity on CT findings or the DoD classification. The lack of observed association between GCL changes and TBI severity and CT findings may suggest patchy RGC loss after TBI, as may be seen in diabetes mellitus, sometimes affecting the peripheral retina only, or that macular RGC may be relatively spared in TON, as seen in other diseases such as glaucoma^42,43^.

RNFL thickness changes are detectable through OCT as early as 2 weeks after TBI, with atrophy developing by 6 weeks^25,36,44^. Importantly we report significant ongoing RNFL thinning in patients seen 42–150 days after injury, with continued (lesser) thinning observed even after 150 days. These findings are consistent with previous reports of late retinal neurodegeneration, which occurs years after TBI, and the well-described long-term risk of dementia^19,45,46^. Given the relatively small magnitude of observed changes, OCT may therefore contribute to objective tools for detecting TBI and assessing its severity as part of a multimodal assessment, but further work should also investigate the extent to which initial RNFL and GCL thinning or thickening, as well as subsequent progressive retinal neurodegeneration, relate to the long-term dementia risk.

The study has important limitations, including the lack of follow up data on over 50% of the cohort. The patients with follow up data represent those with more severe injury, potentially causing over-estimation of the frequency of OCT changes and visual symptoms, although this does not affect the observed association between OCT changes and injury severity, or the baseline assessments. In addition, because patients were not recruited to a protocolised study, the duration of follow up varied, which we addressed by assessing OCT change over time, but which could still mean that transient changes, such as initial thickening with subsequent thinning, may be missed.

In conclusion, we report clinically detectable OCT changes in 50% of patients with TBI of a degree which associates with injury severity as assessed by the DoD criteria and Marshall scoring of CT imaging. Ophthalmologists assessing patients with TBI should expect to see subclinical OCT changes.

## Supplementary Information

Supplementary Information.

## Data Availability

Raw data were generated at and are owned by University Hospitals Birmingham NHS Foundation Trust. Derived data supporting the findings of this study are available from the corresponding author on reasonable request, subject to data transfer agreement.

## Declarations

### Institutional approval

Prospective data on TBI patients were collected as part of a service evaluation registered with the Clinical Governance Department and data analysis for this research study was approved by the Health Research Agency (23/HRA/0136) and the Research and Development Department at University Hospitals Birmingham NHS Foundation Trust.
