# Supplementary material for: Retinal manifestations of traumatic brain injury
Source: Sci Rep. 2025 Apr 29;15:14992. doi: 10.1038/s41598-025-94091-1 (PMC12041581; doi:10.1038/s41598-025-94091-1)
Supplement: Supplementary file 1 — Supplementary Information. [file 41598_2025_94091_MOESM1_ESM.pdf]

Supplementary Table S1: DoD and Marshall CT head classification. LOC loss of consciousness, PTA post-traumatic amnesia, GCS Glasgow coma score

Classification	Score	Criteria
**DoD Classification [7]**	Mild	● normal structural imaging<br>● LOC 0 to 30 minutes<br>● alteration of consciousness/mental state a moment up to 24 hours<br>● PTA 0 to 1 day<br>● GCS in the first 24 hours after injury 13 to 15
	Moderate	● normal or abnormal structural imaging<br>● LOC >30 minutes to 24 hours<br>● PTA >1 day to <7 days<br>● GCS in the first 24 hours after injury 9 to 12
	Severe	● normal or abnormal structural imaging<br>● LOC >24 hours<br>● alteration of consciousness/mental state > 24 hours<br>● PTA >7 days<br>● GCS in the first 24 hours after injury <9
**Marshall CT head classification [9]**	I: Diffuse injury (no visible pathology)	● no visible intracranial pathology
	II: Diffuse injury	● midline shift of 0 to 5 mm<br>● basal cisterns remain visible.<br>● no high or mixed density lesions >25cm^3
	III: Diffuse injury (swelling)	● midline shift of 0 to 5 mm<br>● basal cisterns compressed or completely effaced)<br>● no high or mixed density lesions >25cm^3
	IV: Diffuse injury (midline shift)	● midline shift of >5 mm<br>● no high or mixed density lesions >25cm^3
	V: Evacuated mass lesion	● any lesion evacuated surgically
	VI: Non-evacuated mass lesion	● high or mixed density lesions >25cm^3<br>● not surgically evacuated

Supplementary Table S2: Summary of RNFL changes in patients with follow up. (n=62)

OCT classification	Number of patients (%)	Patients with GCL changes (thinning or thickening) (%)	TBI severity (Marshall Score)				TBI severity (DoD classification)		
			I	II	≥III	No CT	Mild	Moderate	Severe
**TON**	2 (3.2)	2 (100)	0	2	0	0	0	1	1
**Single sector RNFL thinning**	4 (6.5)	2 (50)	1	1	0	2	3	1	0
**Multi-sector RNFL thinning**	13(21)	7 (53.8)	0	9	4	0	0	8	5
**RNFL thickening only**	6 (9.7)	1 (16)	1	2	1	2	3	2	1
**GCL changes without RNFL changes**	6 (9.7)	6 (100)	2	3	0	1	1	5	0
**No changes**	31 (50)	0 (0)	2	9	5	15	15	12	4
